# Predictors of failure with high-flow nasal oxygen therapy in COVID-19 patients with acute respiratory failure: a multicenter observational study

**DOI:** 10.1186/s40560-021-00538-8

**Published:** 2021-03-05

**Authors:** Ricard Mellado-Artigas, Luis Eduardo Mujica, Magda Liliana Ruiz, Bruno Leonel Ferreyro, Federico Angriman, Egoitz Arruti, Antoni Torres, Enric Barbeta, Jesús Villar, Carlos Ferrando, for the COVID-19 Spanish ICU Network, Marina Vendrell, Gerard Sánchez-Etayo, Amalia Alcón, Isabel Belda, Mercé Agustí, Albert Carramiñana, Isabel Gracia, Miriam Panzeri, Irene León, Jaume Balust, Ricard Navarro, María José Arguís, María José Carretero, Cristina Ibáñez, Juan Perdomo, Antonio López, Jorge Mejia, Manuel López-Baamonde, Tomás Cuñat, Marta Ubré, Antonio Ojeda, Andrea Calvo, Eva Rivas, Paola Hurtado, Roger Pujol, Nuria Martín, Javier Tercero, Pepe Sanahuja, Marta Magaldi, Miquel Coca, Elena del Rio, Julia Martínez-Ocon, Paula Masgoret, Monserrat Tio, Angel Caballero, Raquel Risco, Raquel Bergé, Lidia Gómez, Nicolás de Riva, Ana Ruiz, Beatriz Tena, Sebastián Jaramillo, José María Balibrea, Francisco Borja de Lacy, Ana Otero, Ainitze Ibarzabal, Raquel Bravo, Anna Carreras, Daniel Martín-Barreda, Alfonso Jesús Alias, Mariano Balaguer, Jorge Aliaga, Alex Almuedo, Joan Ramón Alonso, Rut Andrea, Gerard Sergi Angelès, Marilyn Arias, Fátima Aziz, Joan Ramon Badía, Enric Barbeta, Toni Torres, Guillem Batiste, Pau Benet, Xavi Borrat, María Borrell, Ernest Bragulat, Inmaculada Carmona, Manuel Castellà, Pedro Castro, Joan Ceravalls, Oscar Comino, Claudia Cucciniello, Clàudia De Peray, Oriol De Diego, Paula De la Matta, Marta Farrero, Javier Fernández, Sara Fernández, Anna Fernández, Miquel Ferrer, Ana Fervienza, María Tallo Forga, Daniel Forné, Clàudia Galán, Andrea Gómez, Eduard Guasch, María Hernández-Tejero, Adriana Jacas, Beltrán Jiménez, Pere Leyes, Teresa López, José Antonio Martínez, Graciela Martínez-Pallí, Jordi Mercadal, Guido Muñoz, José Muñoz, Josep María Nicolás, José Tomás Ortiz, Anna Peiró, Manuel Pérez, Esteban Poch, Margarida Pujol, Eduard Quintana, Bartomeu Ramis, Enric Reverter, Irene Rovira, Pablo Ruiz, Elena Sandoval, Stefan Schneider, Oriol Sibila, Carla Solé, Alex Soriano, Dolors Soy, M. Suárez, Adrián Téllez, Néstor David Toapanta, Antoni Torres, Xavier Urra César Aldecoa, Alicia Bordell, Silvia Martín, Judith Andrés, Alberto Martínez Ruiz, Gonzalo Tamayo Medel, Iñaki Bilbao Villasante, Fernando Iturri Clavero, Covadonga Peralta Álvarez, Julia T. Herrera Díez, Andrea García Trancho, Iñaki Sainz Mandiola, Carmen Ruano Suarez, Angela Ruiz Bocos, Eneritz Urrutia Izagirre, Pablo Ortiz de Urbina Fernández, Naiara Apodaka López, Leire Prieto Molano, Eunate Ganuza Martínez, Iratxe Vallinas Hidalgo, Karmele de Orte Sancho, Celia González Paniagua, Gemma Ortiz Labrador, Mireia Pérez Larrañaga, Marta López Miguelez, Estíbaliz Bárcena Andrés, Erik Urutxurtu Laureano, Maria Jesús Maroño Boedo, Blanca Escontrela Rodríguez, Aitziber Ereñozaga Camiruaga, Deiene Lasuen Aguirre, Ainhoa Zabal Maeztu, Ane Guereca Gala, Iker Castelo Korro, Andrés Álvarez Campo, Alejandro Carcelen Viana, Alejandro Alberdi Enríquez, Xabier Ormazábal Rementeria, Alberto Sánchez Campos, Rosa Gutiérrez Rico, Pablo Barbier Damborenea, Marta Guerenabarrena Momeñe, Borja Cuesta Ruiz, Alejandro López Rico, Ana Rojo Polo, Covadonga García Grijelmo, Mikel Celorrio Reta, Eneko Martín Arroyo, Leire Artaza Aparicio, Iñaki Ituarte Aspiazu, Ane Igeregi Basabe, Itxaso Merino Julian, Isabel Diaz Rico, Maria Paz Martínez, Ramón Adalia Bartolomé, Luigi Zattera, Irina Adalid Hernandez, Leire Larrañaga Altuna, Aina Serrallonga Castells, Adriana Vílchez Garcia, María Núñez, Lorena Román, Isabel Ramos Delgado, Adela Benítez-Cano Martínez, Mireia Chanzá Albert, Juan Carlos Álvarez García, Luis Aguilera Cuchillo, Sandra Beltrán de Heredia, Jesús Carazo Cordobés, Carlos Alberto García Bernedo, Fernando Escolano Villén, Francisco Javier Redondo Calvo, Rubén Villazala González, Victor Baladron González, Patricia Faba, Omar Montenegro, Natalia Bejarano Ramírez, Sergio Marcos Contreras, Alejandro Garcia Rodríguez, Saleta Rey Vázquez, Cristina Garcia Pérez, Eva Higuera Miguelez, Irene Pérez Blanco, David García Rivera, Ane Martín de la Fuente, Marta Pardo, Vanessa Rodriguez, Unai Bengoetxea, Fernando Ramasco, Sheila Olga Santidrián Bernal, Alvar Santa Cruz Hernando, Antonio Planas Roca, Carlos Figueroa Yusta, Esther García Villabona, Carmen Vallejo Lantero, Eva Patiño Rodriguez, Alvaro Esquivel Toledo, David Arribas Méndez, Mar Orts Rodriguez, Rosa Méndez Hernández, Jesús Nieves Alonso, Inés Imaz Artazcoz, Sonia Expósito Carazo, Carlos Román Guerrero, Elena Rojo Rodríguez, Ricardo Moreno González, Julia Hernando Santos, Jara Torrente Pérez, Esperanza Mata Mena, Manuel José Muñoz Martínez, Enrique Alday Muñoz, Patricia Martin Serrano, Laura Cotter Muñoz, Amadea Mjertan, Diego Gutierrez Martínez, Carmen Rodríguez García, Olaya Alonso Viejo, Juan Alvarez Pereira, Ana Carmona Bonet, Diana Parrado López, Eva de Dios Tomas, Rafael Martín Celemin, María Luisa Meilan Paz, Luis Quecedo Gutiérrez, Noemí Diaz Velasco, Gabriel Martin Hernández, Francisco Garcia del Corral, Gloria Hernandez Arias, David Rodriguez Cuesta, Ana Gómez Rice, Encarna Mateos Sevillano, Natalia Olmos Molpeceres, Beatriz Domínguez, Ana Vázquez Lima, Ángel Candela, Ismael A. Acevedo Bambaren, Maria Isabel Albala Blanco, Paloma Alonso Montoiro, Fernando Álvarez Utrera, Juan Avellanosa Esteruelas, Amal Azzam López, Alberto José Balvis Balvis, Tommaso Bardi, María Beltrán Martín, Jacobo Benatar Haserfaty, Alberto Berruezo Camacho, Laura Betolaza Weimer, María del Mar Carbonell Soto, Cristina Carrasco Seral, Cristina Cerro Zaballos, Elizabeth Claros Llamas, Pilar Coleta Orduna, Ingrid P. Cortes Forero, Pascual Agustín Crespo Aliseda, María Angélica de Pablo Pajares, Yolanda Díez Remesal, Trinidad Dorado Díaz, Noemí Echevarría Blasco, María Elena Elías Martín, Javier Felices Triviño, Natalia Fernández López, Cristina Fernández Martín, Natalia Ferreiro Pozuelo, Luis Gajate Martín, Clara Gallego Santos, Diego Gil Mayo, María Gómez Rojo, Claudia González Cibrián, Elena Herrera López, Borja Hinojal Olmedillo, Berta Iglesias Gallego, Sassan Khonsari, María Nuria Mane Ruiz, María Manzanero Arroyo, Ana María Mariscal Ortega, Sara Martín Burcio, María del Carmen Martín González, Ascensión Martín Grande, Jose Juan Martín López, Cecilia Martín Rabes, Marcos Martínez Borja, Nilda Martínez Castro, Adolfo Martínez Pérez, Snejana Matcan, Cristina Medrano Viñas, Lisset Miguel Herrera, Adrián Mira Betancur, María Montiel Carbajo, Javier Moya Moradas, Lorena Muñoz Pérez, Mónica Nuñez Murias, Eva Ordiales González, Óscar Ordoñez Recio, Miguel Ángel Palomero Rodriguez, Diego Parise Roux, Lucia Pereira Torres, David Pestaña Lagunas, Juana María Pinto Corraliza, Marian Prieto Rodrigo, Inmaculada Rodriguez Diaz-Regaño, David Rodriguez Esteban, Víctor Rojas Pernia, Álvaro Ruigómez Saiz, Bárbara Saavedra Villarino, Noemí Samaranch Palero, Gloria Santos Pérez, Jaume Serna Pérez, Ana Belén Serrano Romero, Jesús Tercero López, Carlos Tiscar García, Marta de la Torre Concostrina, Eva María Ureta Mesa, Eva Velasco Olarte, Judith Villahoz Martínez, Raúl Villalaba Palacios, Gema Villanueva García, Cristina Vogel de Medeiros, Soraya Gholamian Ovejero, Marta Vicente Orgaz, Patricia Lloreda Herradon, Cristina Crespo Gómez, Tatiana Sarmiento-Trujillo, Noemí García Medina, María Martínez García, Carles Espinós Ramírez, Nabil Mouhaffel Rivero, Jose Antonio Bernia Gil, Sonsoles Martín, María Victoria Moral, Josefina Galán, Pilar Paniagua, Sergio Pérez, Albert Bainac, Ana Arias, Elsa Ramil, Jorge Escudero, Pablo Monedero, Carmen Cara, Andrea Lara, Elena Mendez Martínez, Jorge Mendoza, Íñigo Rubio Baines, Carmen Sala Trull, Pablo Montero López, Alfredo Gea, Alejandro Montero, Rocío Armero Ibañez, Juan Vicente Llau Pitarch, Fernando Rauer Alcóver, Cristina Álvarez Herreros, Cyntia Sánchez Martín, Lucía López Ocáriz Olmos, Marta Navas Moruno, M. F. Fernando García Montoto., Mirón Rodriguez, Laura Fuentes Coco, Cristina Hernández Gamito, Antonio Barba Orejudo, Luis Gerardo Smith Vielma, Yasmina González Marín, Francisco de Borja Amador Penco, Marta Donoso Domínguez, Silvia Esquivel Ramírez, José Antonio Carbonell, Berta Monleón López, Sara Martínez-Castro, Gerardo Aguilar, María Gestal, Pablo Casas, Angel Outeiro Rosato, Andrea Naveiro Pan, María Alonso Portela, Adrián García Romar, Eva Mosquera Rodríguez, Diego Ruanova Seijo, Pablo Rama Maceiras, Francisco Castro-Ceoane, Esther Moreno López, Sergio Gil, Julia Guillén Antón, Patricia García-Consuegra Tirado, Aurora Callau Calvo, Laura Forés Lisbona, María Carbonell Romero, Belén Albericio Gil, Laura Pradal Jarne, María Soria Lozano, Diego Loscos López, Andrea Patiño Abarca, Jordi Serrano, Javier Pérez-Asenjo, Ángel Díez-Domínguez, Ion Zubizarreta, Jon Ramos, Iosu Fernández, Emilio Maseda, Alejandro Suárez de la Rica, Javier Veganzones, Itziar Insausti, Javier Sagra, Sofía Díaz Carrasco, Ana Montero Feijoo, Julio Yagüe, Ignacio Garutti, Javier Hortal, Patricia Piñeiro, Matilde Piñeiro, Matilde Zaballos, Jamil Cedeño, Pablo García-Olivares, Alberto Garriido, Jose Eugenioi Guerrero, Eva Bassas Parga, Carmen Deiros Garcia, Elisenda Pujol Rosa, Ana Tejedor Navarro, Roser Font Gabernet, Maria José Bernat, Meritxell Serra Valls, Cristina Cobaleda Garcia-Bernalt, Jesus Fernanz Anton, Adriana Aponte Sierra, Lucia Gil Gomez, Olaia Guenaga Vaqueiro, Susana Hernandez Marin, Laura Pardo Pinzon, Sira Garcia Aranda, Carlos Briones Orejuela, Edgar Cortes Sánchez, Alejandro Romero Fernández, Esther Fernández SanJosé, Patricia Iglesias Garsabal, Guillermo Isidro Lopez, Ana Vicol, Sara Espejo Malagon, María Sanabra Loewe, Laura Grau Torradeflo, Lourdes Blanco Alcaide, Gloria Buenaventura Sanclemente, Pere Serra Pujol, Gustavo Cuadros Mendoza, Miroslawa Konarska, Fedra Bachs Almenara, Agnieszka Golska, Aleix Carmona Blesa, Arantxa Mas Serra, Javier Ripolles Melchor, Ana Nieto Moreno, Káteri Chao Novo, Sandra Gadín López, Elena Nieto Moreno, Bérénice Gutiérrez Tonal, Elena Lucena de Pablo, Barbara Algar Yañez, Beatriz Vázquez Rivero, Beatriz Nozal Mateo, Marina de Retes, Norma Aracil Escoda, Cristina Gallardo Mayo, Rosa Sanz González, Alicia Ruiz Escobar, Maria Laura Pelegrina López, Marina Valenzuela Peña, David Stolle Dueñas, Ane Abad Motos, Alfredo Abad-Gurumeta, Ana Tirado Errazquin, Elena Sáez Ruiz, Nerea Gómez Pérez, Francisco de Borja Bau González, Cesar Morcillo Serra, Jessica Souto Higueras, Rosario Vicente, Raquel Ferrandis, Silvia Polo Martín, Azucena Pajares Moncho, Ignacio Moreno Puigdollers, Juan Pérez Artacho Cortés, Ana Moret Calvo, Ana Pi Peña, María Catalán Fernández, Marina Varela, Pilar Díaz Parada, Raquel Rey Carlín, Sarra Barreiro Aragunde, María Isabel Forés Chiva, A. Javier Agulló, Antonio Pérez Ferrer, María Galiana, Antoni Margarit, Válerie Mourre del Rio, Eva Heras Muxella, Anna Vidal

**Affiliations:** 1 Department of Anesthesiology and Critical Care, Hospital Clínic, Institut D’investigació August Pi i Sunyer, Villarroel 170, 08025 Barcelona, Spain; 2 Department of Mathematics, Faculty of Engineering, Universitat Politècnica de Catalunya, Barcelona, Spain; 3 Interdepartmental Division of Critical Care Medicine, University of Toronto, Toronto, Canada; 4 Department of Critical Care Medicine, Sunnybrook Health Sciences Centre, Toronto, Canada; 5 Ubikare Technology, Vizcaya, Spain; 6 Department of Respirology, Hospital Clínic, Institut D’investigació August Pi i Sunyer, Barcelona, Spain; 7 CIBER de Enfermedades Respiratorias, Instituto de Salud Carlos III, Madrid, Spain; 8 Multidisciplinary Organ Dysfunction Evaluation Research Network, Research Unit, Hospital Universitario Dr. Negrin, Las Palmas de Gran Canaria, Spain; 9 Keenan Research Center at the Li Ka Shing Knowledge Institute, St Michael’s Hospital, Toronto, Ontario Canada

**Keywords:** High-flow nasal oxygen therapy, COVID-19, Invasive mechanical ventilation, Hypoxemic respiratory failure

## Abstract

**Purpose:**

We aimed to describe the use of high-flow nasal oxygen (HFNO) in patients with COVID-19 acute respiratory failure and factors associated with a shift to invasive mechanical ventilation.

**Methods:**

This is a multicenter, observational study from a prospectively collected database of consecutive COVID-19 patients admitted to 36 Spanish and Andorran intensive care units (ICUs) who received HFNO on ICU admission during a 22-week period (March 12-August 13, 2020). Outcomes of interest were factors on the day of ICU admission associated with the need for endotracheal intubation. We used multivariable logistic regression and mixed effects models. A predictive model for endotracheal intubation in patients treated with HFNO was derived and internally validated.

**Results:**

From a total of 259 patients initially treated with HFNO, 140 patients (54%) required invasive mechanical ventilation. Baseline non-respiratory Sequential Organ Failure Assessment (SOFA) score [odds ratio (OR) 1.78; 95% confidence interval (CI) 1.41-2.35], and the ROX index calculated as the ratio of partial pressure of arterial oxygen to inspired oxygen fraction divided by respiratory rate (OR 0.53; 95% CI: 0.37-0.72), and pH (OR 0.47; 95% CI: 0.24-0.86) were associated with intubation. Hospital site explained 1% of the variability in the likelihood of intubation after initial treatment with HFNO. A predictive model including non-respiratory SOFA score and the ROX index showed excellent performance (AUC 0.88, 95% CI 0.80-0.96).

**Conclusions:**

Among adult critically ill patients with COVID-19 initially treated with HFNO, the SOFA score and the ROX index may help to identify patients with higher likelihood of intubation.

**Supplementary Information:**

The online version contains supplementary material available at 10.1186/s40560-021-00538-8.

## Background

The novel coronavirus 2019 (COVID-19) infection has spread worldwide causing thousands of cases of acute respiratory failure with an associated high mortality rate [1, 2]. Critically-ill patients with COVID-19 often have profound hypoxemia which may partially explain the extremely high use of invasive ventilatory support for long periods of time shown in these subjects [3, 4]. This issue, combined with the sharp rise in the incidence of this disease, has led to an unprecedented pressure on many healthcare systems and hospitals worldwide [4–7].

High-flow nasal oxygen (HFNO) reduces the need for endotracheal intubation in patients with acute respiratory failure [8–10]. In the last few months, several studies have reported experiences with HFNO therapy in patients with COVID-19 [11, 12]. Also, a recent publication suggested that HFNO compared to oxygen therapy could decrease the requirements for invasive mechanical ventilation in these patients [13]. If validated, the use of HFNO would not only be beneficial for individual patients treated noninvasively but also to those planned for invasive mechanical ventilation through the rational allocation of resources. Conversely, delaying intubation by choosing a non-invasive approach may be associated with worse outcomes in patients with the acute respiratory distress syndrome (ARDS) [3, 14–16]. Therefore, identifying those at higher risk of failure could be highly valuable for avoiding delays in choosing the best management approach.

In this study, we sought to describe the use of HFNO in adult patients with COVID-19 acute respiratory failure and to identify factors associated with a greater risk of intubation. We also aimed to derive a parsimonious predictive score for intubation as an aid in daily clinical decision-making.

## Material and methods

### Study design and setting

We conducted a prospective, multicenter, cohort study of consecutive patients with COVID-19 related acute respiratory failure admitted to 36 hospitals from Spain and Andorra (see Supplementary file) [17]. The study was approved by the referral Ethics Committee of Hospital Clínic, Barcelona, Spain (code #HCB/2020/0399) and was conducted according to the amended Declaration of Helsinki. This report follows the “Strengthening the Reporting of Observational Studies in Epidemiology (STROBE)” guidelines for observational cohort studies [18]. Gathering of data is ongoing and as of August 13, a total of 1129 patients were included.

### Study population

For the present study, all consecutive patients included in the database from March 12 to August 13, 2020 that fulfilled the following inclusion criteria were analyzed: age ≥18 years, ICU admission with a diagnosis of COVID-19 related acute respiratory failure, positive confirmatory nasopharyngeal or pulmonary tract sample, and HFNO initiated on ICU admission day. Exclusion criteria were the use of oxygen therapy and non-invasive or invasive mechanical ventilation prior to HFNO or the absence of data regarding respiratory management on day 1 after ICU admission.

### Data collection

Patients’ characteristics were collected prospectively from electronic medical records by physicians trained in critical care according to a previously standardized consensus protocol. Each investigator had a personal username/password, and entered data into a specifically pre-designed online data acquisition system (CoVid19.ubikare.io) endorsed and validated by the Spanish Society of Anesthesiology and Critical Care (SEDAR) [19]. Patient confidentiality was protected by assigning a de-identified code. Recorded data included demographics [age, gender, body mass index (BMI)], comorbidities and disease chronology [time from onset of symptoms and from hospital admission to initiation of respiratory support, ICU length of stay], vital signs [temperature, mean arterial pressure, heart rate], laboratory parameters (blood test, coagulation, biochemical), ratio of oxygen saturation to inspired oxygen fraction, divided by respiratory rate (ROX) index, and severity scores such as the Sequential Organ Failure Assessment (SOFA) and Acute Physiology and Chronic Health Evaluation II (APACHE II) scores. Data regarding physiological parameters was collected once daily. Site investigators collected what they considered to be the most representative data of each day from ICU admission to ICU discharge. After ICU discharge, patients were followed-up until hospital discharge.

### Study outcomes

The primary outcome was the assessment of factors at ICU admission (ICU day 1) associated with the need for endotracheal intubation up to 28 days after HFNO initiation. The decision to intubate was made at the discretion of the attending physician at each participating site. Secondary goals were the development of a predictive model to estimate the probability of endotracheal intubation after HFNO and the assessment of between-center variability in the likelihood of receiving intubation after HFNO had been started.

### Statistical analysis

We used descriptive statistics to summarize patients’ baseline characteristics. We compared the baseline characteristics of patients who required intubation with those who did not require intubation. Specifically, continuous variables were compared with the *T* test with unequal variances or the Mann-Whitney *U* test, as appropriate. Categorical variables were compared using the chi-square tests or Fisher’s exact test as appropriate. In order to identify factors associated with the likelihood of intubation, we fit a multivariable logistic regression model with endotracheal intubation as the dependent variable. A priori selected variables were those considered of clinical relevance as well as variables that were significantly associated with the outcome in the bivariate analysis (at a *p* value threshold of 0.2 or less). We report odds ratios (OR) with their associated 95% confidence intervals (CI).

Then, we sought to derive a parsimonious predictive model for intubation among patients treated with HFNO on the first day of ICU admission. Thus, we randomly split the full dataset in two parts: (1) a training dataset including 70% of the patients, and (2) a validation dataset including the remaining 30% of subjects. In the derivation step, all variables showing statistical significance with the outcome were chosen, and a final model based on the best accuracy was selected after performing tenfold cross-validation. The final model calibration was tested in the split validation cohort with the use of the Brier score. A receiver operating characteristic (ROC) curve was constructed to display the area under the curve (AUC) for the predictive model. The optimal cutoff was considered as the one showing the best accuracy. At this cutoff, the performance of the model is presented as sensitivity, specificity, positive and negative predictive values, and positive and negative likelihood ratios and their accompanying 95% CI. An online calculator is shown to estimate the likelihood of HFNO failure for each individual patient. Since validation datasets with few observations can provide imprecise estimates of performance, a sensitivity analysis to assess final model performance using enhanced bootstrapping was also carried out [20].

Additionally, since one of the goals of the present study was to assess center-related variability regarding the clinical decision to intubate, a mixed-effects multivariable logistic regression was fit as a secondary analysis. We fit a logistic model with a random intercept (for each center that recruited more than 10 patients), to account for possible correlation and differences in the baseline risk of intubation based on practice variation between sites. The proportion of variance explained by all fixed factors is presented as the marginal *R*^2^ and the proportion of variance explained by the whole model is presented as the conditional *R* [2, 21].

To account for missing data, which occurred in 6% of the observations of interest, we performed multiple imputation based on Markov chain Monte Carlo methods [22]. Specifically, for regression analysis, we removed subjects with extensive missing data (>50%). Briefly, for every missing value, we created 5 matrices, each one with 1000 imputations. Final imputed values for each missing observation were calculated as the median of all imputations. Imputation of the dependent variable (intubation) was not performed. We used a threshold of 0.05 for statistical significance and all reported tests are two-sided. For statistical analysis, we used the R software (R Foundation for Statistical Computing, Vienna, Austria) and included mice, lme4, caret, OptimalCutpoints, performance, and pROC packages.

## Results

**Fig. 1:**
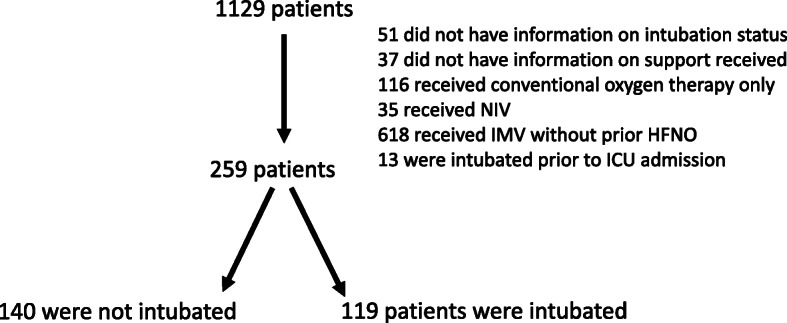
Patient flowchart. Two hundred fifty-nine patients were included and followed up until ICU discharge or death. NIV, non-invasive ventilation; IMV, mechanical ventilation

From March 12 to August 13, 2020, 259 critically ill patients with COVID-19 related acute respiratory failure were initially treated with HFNO and were included in the present study (Fig. 1). From those, 140 (54.0%) patients were intubated and mechanically ventilated after ICU admission, of whom 74 patients (52.9%) were intubated on the ICU admission day. SOFA score and APACHE II were higher in patients requiring intubation while respiratory rate, PaO_2_/FiO_2_ ratio, and ROX index were lower (Table 1).

**Table 1:** Baseline characteristics of 259 patients with COVID-19 acute respiratory failure

	ALL (***n***=259)	Intubation		***P*** value^**1**^
		NO (***n***=119)	YES (***n***=140)	
**Patients demographics and comorbidities**				
Age, years	62 (55-70)	62 (53-69)	63 (55-70)	0.39
Gender, male	185 (71%)	90 (76%)	95 (68%)	0.16
BMI, kg/m^2^	28 (25-32)	27 (25-31)	28 (25-32)	0.11
Number of comorbidities	1 (0-2)	1 (0-2)	1 (0-1)	0.73
Hypertension	109 (42%)	47 (420%)	62 (44%)	0.45
Ischemic heart disease	15 (6%)	8 (7%)	7 (5%)	0.60
Diabetes	45 (17%)	20 (16%)	25 (18%)	0.87
Chronic respiratory disease	14 (5%)	6 (5%)	8 (6%)	1
Chronic kidney disease	22 (8%)	10 (8%)	12 (9%)	1
Malignancy	12 (4%)	9 (8%)	3 (2%)	0.07
Days from hospital to ICU admission.	2 (1-4)	3 (1-4)	2 (0-4)	<0.01
**Scores**				
SOFA	4 (3-7)	4 (3-5)	6 (4-8)	<0.01
Non-respiratory SOFA	1 (0-3)	0 (0-1)	3 (0-4)	<0.01
APACHE II	11 (8-16)	9 (6-13)	13 (10-18)	<0.01
Glasgow Coma Scale	15 (15-15)	15 (15-15)	15 (15-15)	0.03
**Vital signs**				
Respiratory rate, rpm	26 (22-30)	24 (20-28)	29 (25-34)	<0.01
Heart rate	80 (72-93)	78 (70-90)	84 (75-95)	0.01
SBP, mmHg	126 (22)	126 (18)	125 (23)	0.33
SpO_2_, %	90 (87-93)	91 (89-94)	89 (85-92)	<0.01
PaO_2_/FiO_2_	109 (83-151)	124 (95-166)	96 (78-144)	<0.01
ROX index	4.4 (3.3-6.1)	5.4 (5.1-7.3)	3.5 (3.2-5.1)	<0.01
pH	7.45 (7.40-7.47)	7.46 (7.44-7.49)	7.40 (7.39-7.40)	<0.01
PaCO_2_, mmHg	36 (31-41)	34 (31-38)	36 (30-47)	0.01
**Laboratory findings**				
Creatinine, mg/dL	0.8 (0.7-1.1)	0.8 (0.7-1)	0.9 (0.7-1.2)	0.32
Leucocyte count, 10^9^/μL	7.5 (5.6-11.5)	7.1 (5.8-10.8)	7.7 (5.6-12.3)	0.16
Platelet count, 10^12^/ μL	235 (175-319)	248 (197-342)	234 (164-300)	0.03
D-dimer, U/L	985 (600-2200)	915 (600-1970)	1100 (680-2620)	0.12

Continuous covariates are shown as mean (SD) or median (IQR). Categorial variables are presented as *n* (%)

^1^Means are compared with the Student’s *T* test, medians with Mann-Whitney *U* test and proportions with either the Chi^2^ or Fisher exact test. ROX index was calculated as [(SpO_2_/inspired oxygen fraction)/respiratory rate (RR)]

*BMI* body mass index; *ICU* intensive care unit; *SOFA* Sequential Organ Failure Assessment; *APACHE* Acute Physiology and Chronic Health Evaluation II; *SBP* systolic blood pressure; *SpO*_*2*_ peripheral oxyhemoglobin saturation; *PaO*_*2*_*/FiO*_*2*_ partial pressure of arterial oxygen to inspiratory oxygen fraction ratio; *PaCO*_*2*_ partial pressure of carbon dioxide

### Associated factors and predictive model for intubation

**Fig. 2:**
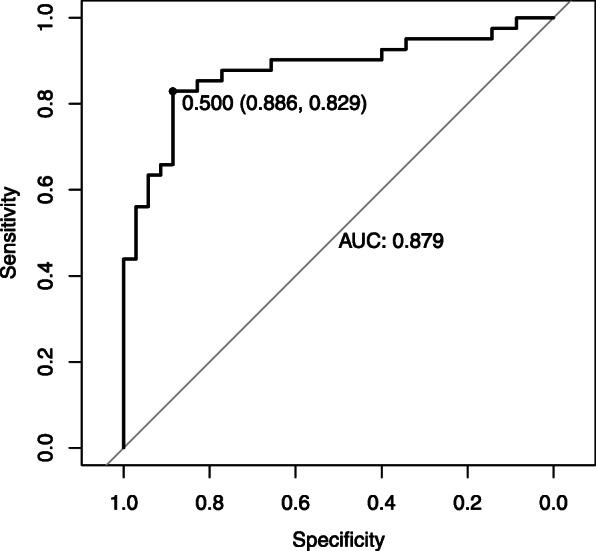
ROC curve in the validation dataset. AUC, area under the curve. The black dot on the ROC curve depicts the optimal threshold, as defined as the probability cut-off with the best accuracy

After excluding 3 subjects for extensive missing data, 256 patients were included in the multivariable logistic regression analysis. Baseline non-respiratory SOFA score (OR 1.78; 95% CI 1.41-2.35), ROX index (OR 0.53; 95% CI 0.38-0.72), and pH (OR 0.47; 95% CI: 0.24-0.86) were associated with the need for intubation (Table 2). A model including the non-respiratory SOFA, the ROX index and cancer showed the best accuracy in the training dataset (see Additional file 1, Table S1). However, given that cancer was a protective factor for intubation, which probably meant treatment escalation limitation, a simpler model including non-respiratory SOFA and the ROX index was selected. In the validation subset, this model had excellent calibration (Brier score of 0.14) and discrimination (AUC of 0.88, 95% CI 0.80-0.96) (see Table 3 and Fig. 2).

**Table 2:** Associated factors with intubation in 256 patients with COVID-19 treated with HFNO

	Odds ratio (95% CI)	***P*** value^**1**^
Non-respiratory SOFA score	1.78 (1.41-2.35)	<0.01
ROX index	0.53 (0.38-0.72)	<0.01
pH, per 0.1-unit increase	0.47 (0.24-0.86)	0.03
Leucocyte count, 10^9^/μL	1.07 (1.001-1.13)	0.01
Malignancy	0.14 (0.02-0.88)	0.04
BMI, kg/m^2^	1.05 (0.97-1.14)	0.23
PaO_2_/FiO_2_ (per 10-point increase)	1.04 (0.97-1.11)	0.21
Gender (female)	1.60 (0.73-3.54)	0.24
D-dimer, U/L	1.00 (0.99-1.00)	0.31
APACHE II	0.95 (0.87-1.04)	0.26
Glasgow Coma Scale	0.51 (0.10-1.08)	0.34
Respiratory rate, rpm	0.97 (0.90-1.04)	0.50
Heart rate, bpm (per 10-bpm increase)	1.08 (0.87-1.35)	0.46
Time from symptom onset to ICU admission (per 1-day increase)	1.02 (0.95-1.12)	0.64
SBP, mmHg (per 10-mmHg increase)	0.96 (0.80-1.14)	0.66
PaCO_2_, mmHg (per 5-mmHg increase)	1.03 (0.87-1.21)	0.76

^1^Based on a multivariable logistic regression model after multiple imputation, 2 subjects were excluded for extensive missing data (>50% variables)

*CI* confidence interval; *HFNO* high flow nasal oxygen treatment; *SOFA* Sequential Organ Failure Assessment; *SBP* systolic blood pressure; *BMI* body mass index; *SpO*_*2*_ peripheral oxyhemoglobin saturation; *ICU* intensive care unit; *PaO*_*2*_*/FiO*_*2*_ partial pressure of arterial oxygen to inspiratory oxygen ratio; *APACHE* Acute Physiology and Chronic Health Evaluation II; *PaCO*_*2*_ partial pressure of carbon dioxide; *AIC* Akaike information criterion

**Table 3:** Discrimination ability of the model in the test dataset using a 50% probability-of-intubation cut-off

Parameter	Value (95% CI)
Sensitivity	83% (68-91%)
Specificity	89% (74-95%)
Positive predictive value	89% (76-96%)
Negative predictive value	82% (67-91%)
Positive likelihood ratio	7.3 (2.9-18.4)
Negative likelihood ratio	0.20 (0.09-0.38)

*CI* confidence interval

Additionally, 216 patients, enrolled in 7 centers with 10 or more cases, were included in a mixed-effect analysis (see Additional file 1, Table S2). Baseline non-respiratory SOFA score and ROX index remained as independent predictors of intubation (see Additional file 1, Table S2). Overall, fixed effects explained 63% of the variability of the outcome while individual centers explained an additional 1% (see Additional file 1, Table S3 and Figure S1). An online calculator to predict the likelihood of intubation given baseline non-respiratory SOFA score and ROX index was developed (see https://desbancar.shinyapps.io/DESBANCAR/).

Out-of-sample model performance using enhanced bootstrapping is shown in the supplementary file (“Further details on statistical analysis,” “Results,” and “Figure S2”).

## Discussion

In this multicenter cohort study of 259 critically ill adult patients with COVID-19 initially treated with HFNO, the need for intubation and invasive mechanical ventilation was frequent and occurred in more than 50% of patients. Non-respiratory SOFA and the ROX index were the main predictors of endotracheal intubation.

Unlike previous studies in non-COVID patients [9, 23], poor oxygenation at baseline, as measured by PaO_2_/FiO_2_, was not a reliable predictor of intubation. While hypoxemia seems often homogenously noticeable in this population, its mechanisms may be multifactorial and might change over time as the disease progresses [24]. Cressoni et al. described the distinction between anatomic to functional shunt in ARDS, and Gattinoni et al. have recently reported that the ratio of the shunt fraction to the gasless compartment in COVID-19 subjects is often higher than the values found in ARDS [25, 26]. Recently, Chiumello et al. highlighted the differential radiologic pattern of COVID-19 patients as compared to non-COVID-19 ARDS [27]. Similar to previous studies in both non-COVID and COVID patients, our study supported how ROX index, which encompasses information from both oxygenation and respiratory rate, was useful to predict intubation [12, 28]. In the absence of non-pulmonary involvement, a ROX index of 3.5 at admission conferred a 50% chance of intubation, which was 83% sensitive and 89% specific for HFNO failure. Of note, the present study differs from previous reports in the percentage of patients receiving HFNO from the total population of patients with COVID-19 related acute respiratory failure [5, 6]. Specifically, the patient population in the present study comprised 24% of the whole database, potentially showing that clinicians seemed to be keener (compared to previously published reports) on using this non-invasive oxygenation strategy in this patient population. This in turn may also explain the lower PaO_2_/FiO_2_ ratios that were often observed [5, 6] and potentially, the lack of impact on the initial decision to switch from HFNO to invasive mechanical ventilation. Although high-quality evidence is needed to assess the effect of HFNO in COVID-19 patients, its use has increased since the start of the pandemic [29]. Moreover, recently published observational data suggests HFNO might increase ventilator-free days and decrease ICU length of stay without incurring in excessive mortality [10].

Our parsimonious model, which included non-respiratory SOFA and the ROX index, to predict intubation among patients with COVID-19 treated with HFNO showed excellent discrimination and may be helpful in the decision-making process at the bedside. The model also shows strong clinical rationale. It is plausible that as lung mechanics deteriorated in some patients, respiratory drive increased, making the ROX index a valuable tool to predict HFNO failure. Likewise, pH was often lower and PaCO_2_ higher in subjects who later became intubated, suggesting fatigue or increased lung injury in failing subjects. Non-respiratory SOFA score was higher in intubated patients and this was mostly related to hemodynamic impairment. Finally, our mixed-effects analysis showed that most of the variability for the need of invasive mechanical ventilation can be explained by baseline factors at admission, while differential “ICU culture” does not appear to play a major role in this decision. This needs to be analyzed in comparison to previous research showing fairly strong center effects, both in the care of patients with septic shock and mechanically ventilated critically ill adults [30, 31].

Our study has several strengths. First, data were collected prospectively in a nationwide project and one of its main goals was to specifically study the relationship between respiratory treatment and outcome. Second, we were able to derive a parsimonious, potentially easy-to-use model that could aid in the identification of patients who may need intubation while being treated with HFNO. However, we acknowledge some limitations of our findings. First, observational studies, especially those multicenter in nature, as our study, are prone to misclassification of relevant covariates and potential predictors. Specifically, physiological parameters were collected once daily, and researchers were instructed to collect the most representative data over the study day. Although unlikely that researchers disregarded the values obtained during HFNO (since they were likely more abnormal than during mechanical ventilation), we cannot ensure completely that some patients, who became intubated on day 1, had their data collected after mechanical ventilation had been started, thus, representing a potential source of bias in the estimation of the predictive model for HFNO failure. Second, missing data on candidate predictors was present in the final sample, rendering our reported associations subject to information bias, and potentially decreasing the precision of our estimates. However, our results were robust while using multiple imputation.

## Conclusions

In conclusion, in this observational study of 259 adult critically ill patients with COVID-19 related acute respiratory failure receiving HFNO, approximately 1 out of 2 patients were intubated during the subsequent ICU stay. Oxygenation at baseline was not a good predictor of HFNO failure, while non-respiratory SOFA, pH, and ROX index were independently associated with intubation. Little variation on the decision to intubate was observed across included centers. Future studies should confirm our findings and evaluate the performance of our model in external cohorts.

## Supplementary Information

**Additional file 1: Table S1:** Final logistic regression model in the training dataset. **Table S2:** Logistic regression in 216 patients from 7 centres with at least 10 cases. **Table S3:** Mixed model, using hospital number as a random variable, in 216 patients from 8 centres with at least 10 cases. **Figure S1**: Effect of centre in the probability of intubation after HFNO. The vertical line depicts the common intercept. Horizontal bars represent 95% confidence interval for each centre. **Figure S2**. Histogram depicting the optimism for each of the 500 models derived in the bootstrapped samples and later validated in the whole cohort.

## Data Availability

The datasets used and/or analyzed during the current study are available from the corresponding author on reasonable request.

## Abbreviations

APACHE: Acute Physiological and Chronic Health disease Classification System
ARDS: Acute respiratory distress syndrome
ARF: Acute respiratory failure
BMI: Body mass index
COVID-19: Coronavirus disease 19
CRP: C-reactive protein
HFNO: High flow nasal oxygen therapy
ICU: Intensive care unit
IQR: Interquartile range
LOS: Length of stay
MV: Mechanical ventilation
NIV: Non-invasive ventilation
PaO2/FiO2: Partial pressure of arterial oxygen to inspiratory oxygen fraction ratio
P-SILI: Patient self-inflicted lung injury
RR: Respiratory rate
SBP: Systolic blood pressure
SOFA: Sequential Organ Failure Assessment
SpO2: Peripheral oxyhemoglobin saturation
STROBE: Strengthening the Reporting of Observational Studies in Epidemiology
V/Q: Ventilation-perfusion
