# Supplementary material for: Predictors of failure with high-flow nasal oxygen therapy in COVID-19 patients with acute respiratory failure: a multicenter observational study
Source: J Intensive Care. 2021 Mar 5;9:23. doi: 10.1186/s40560-021-00538-8 (PMC7934982; doi:10.1186/s40560-021-00538-8)
Supplement: Supplementary file 1 — Additional file 1: Table S1: Final logistic regression model in the training dataset. Table S2: Logistic regression in 216 patients from 7 centres with at least 10 cases. Table S3: Mixed model, using hospital number as a random variable, in 216 patients from 8 centres with at least 10 cases. Figure S1: Effect of centre in the probability of intubation after HFNO. The vertical line depicts the common intercept. Horizontal bars represent 95% confidence interval for each centre. Figure S2. Histogram depicting the optimism for each of the 500 models derived in the bootstrapped samples and later validated in the whole cohort. [file 40560_2021_538_MOESM1_ESM.docx]

**Predictors of failure with high-flow nasal oxygen therapy in COVID-19 patients with acute respiratory failure: a multicentre observational study**

#### *Running title: HFNO failure in COVID-19*

Ricard Mellado-Artigas, MD^1^

Luis Eduardo Mujica, PhD^2^

Magda Liliana Ruiz, PhD^2^

Bruno Leonel Ferreyro, MD^3^

Federico Angriman, MD, MPH^3,4^

Egoitz Arruti, PhD^5^

Antoni Torres, MD, PhD^6,7^

Enric Barbeta, MD^6^

Jesús Villar, MD, PhD^7,8,9^

Carlos Ferrando, MD, PhD^1,7^

for the COVID-19 Spanish ICU Network

From

1. *Department of Anesthesiology and Critical Care, Hospital Clínic, Institut D'investigació August Pi i Sunyer, Barcelona, Spain;*
2. *Department of Mathematics, Faculty of Engineering, Universitat Politècnica de Catalunya;*
3. *Interdepartmental Division of Critical Care Medicine, University of Toronto, Canada;*
4. *Department of Critical Care Medicine. Sunnybrook Health Sciences Centre. Toronto, Canada;*
5. *Ubikare Technology, Vizcaya, Spain;*
6. *Department of Respirology, Hospital Clínic, Institut D'investigació August Pi i Sunyer, Barcelona, Spain;*
7. *CIBER de Enfermedades Respiratorias, Instituto de Salud Carlos III, Madrid, Spain;*
8. *Multidisciplinary Organ Dysfunction Evaluation Research Network, Research Unit, Hospital Universitario Dr. Negrin, Las Palmas de Gran Canaria, Spain;*
9. *Keenan Research Center at the Li Ka Shing Knowledge Institute, St Michael’s Hospital, Toronto, Canada.*

**CORRESPONDING AUTHOR:**

R. Mellado Artigas, MD. Surgical ICU (Department of Anesthesia and Critical Care), Hospital Clinic. Villarroel 170, ZIP code 08025, Barcelona, Spain. Phone number: 0034 932275558. Email: [rmartigas@gmail.com](mailto:rmartigas@gmail.com)

**FURTHER DETAILS ON STATISTICAL METHODS:**

Internal validation with the use of small datasets might be imprecise since performance could largely depend on the split of the sample used for validation. Bootstrapping refers to a statistical method that resamples a study dataset to perform simulations on very different statistical properties. Bootstrapping can be used to estimate the performance of a regression model and be considered more robust than a split dataset approach, especially when the dataset includes a small number of observations^1^. In our study, we performed a sensitivity analysis to estimate out-of-sample accuracy using enhanced bootstrapping.

To do so, the final chosen model, which included non-respiratory SOFA score and ROX index, was assessed in the 256-patient cohort and the area under the curve (AUC) was calculated. AUC here reflects in-sample performance and likely overestimates performance in an external cohort. Second, the whole was resampled 500-times (we kept size at 256 individuals in all samples [“the bootstrapped samples”]). Third, a model with the same variables was developed in each bootstrapped sample. Fourth, each derived model in the bootstrapped samples was validated in the whole 256-patient population. The difference between the performance in each of the bootstrapped samples to the performance in the whole cohort was considered to represent optimism. Fifth, optimism was subtracted from the in-sample AUC, carried out in step one, and mean out-of-sample performance and 95% confidence intervals were computed.

**RESULTS:**

Mean out-of-sample AUC was estimated at 0.84 and 95% CI were defined at 0.78-0.91. Figure S2 shows the distribution of optimism (difference between in-sample accuracy in each bootstrap minus out-of-sample accuracy for each of these models, estimated in the whole cohort).

**Table S1:** Final logistic regression model in the training dataset

| **Variable** | **Beta** | **Standard error** | **P-value** |
| --- | --- | --- | --- |
| Intercept | 1.84 | 0.49 | <0.001 |
| Non-respiratory SOFA score | 0.49 | 0.10 | <0.001 |
| ROX index | -0.51 | 0.10 | <0.001 |

Logistic regression model to assess the likelihood of intubation. Non-respiratory SOFA score, ROX index, and the presence of malignancy all displayed a significant association with intubation in the training dataset, which was positive for non-respiratory score and negative for the ROX index and the presence of malignancy. In order to avoid selecting a variable which had a clear relationship with treatment escalation limitation, malignancy was excluded from the final model. The final model showed an AIC of 190 and an accuracy of 72% in the training dataset which was only slightly worse than the full model (AIC 182, accuracy 74%).

**Table S2:** Logistic regression in 216 patients from 7 centres with at least 10 cases.

|  | **Odds ratio (95% CI)** | **P-value^1^** |
| --- | --- | --- |
| Non-respiratory SOFA score | 1.94 (1.48-2.65) | <0.01 |
| ROX index | 0.54 (0.36-0.77) | <0.01 |
| pH, per 0.1-unit increase | 0.49 (0.24-0.93) | 0.08 |
| Leucocyte count, 10^9^  /μL | 1.03 (0.96-1.12) | 0.36 |
| Malignancy | 0.09 (0.006-1.005) | 0.07 |
| BMI, kg/m^2^ | 1.05 (0.97-1.15) | 0.23 |
| PaO_2_/FiO_2_ (per 10-point increase) | 1.06 (0.97-1.15) | 0.12 |
| Gender (female) | 1.44 (0.60-3.48) | 0.42 |
| D-dimer, U/L | 1.00 (0.99-1.00) | 0.27 |
| APACHE II | 0.93 (0.85-1.04) | 0.21 |
| Glasgow Coma Scale | 0. 37 (0.05-1.22) | 0.26 |
| Respiratory rate, rpm | 0.99 (0.91-1.08) | 0.80 |
| Heart rate, bpm (per 10-bpm increase) | 1.10 (0.86-1.37) | 0.41 |
| Time from symptom onset to ICU admission (per 1-day increase) | 1.01 (0.94-1.12) | 0.79 |
| SBP, mmHg (per 10-mmHg increase) | 0.90 (0.70-1.12) | 0.37 |
| PaCO_2_, mmHg (per 5-mmHg increase) | 1.07 (0.90-1.28) | 0.45 |

^1^Based on a multivariable logistic regression model after multiple imputation. 3 subjects were excluded for extensive missing data (>50% variables). Variables were included if they showed a p-value less than 0.2 in univariate analysis. CI: confidence interval; HFNO: high flow nasal oxygen treatment; SOFA: sequential organ failure assessment; SBP: systolic blood pressure; BMI: body mass index; SpO_2_: peripheral oxyhaemoglobin saturation; ICU: intensive care unit; PaO_2_/FiO_2_: partial pressure of arterial oxygen to inspiratory oxygen ratio; APACHE: Acute Physiology and Chronic Health Evaluation II; PaCO_2_: partial pressure of carbon dioxide; AIC: Akaike information criterion.

**Table S3:** Mixed model, using hospital number as a random variable, in 216 patients from 8 centres with at least 10 cases.

|  | **Odds ratio (95% CI)** | **P-value^1^** |
| --- | --- | --- |
| Non-respiratory SOFA score | 2.00 (1.56-2.55) | <0.01 |
| ROX index | 0.55 (0.40-0.74) | <0.01 |
| pH, per 0.1-unit increase | 0.52 (0.27-1.004) | 0.05 |
| PaO_2_/FiO_2_ (per 10-point increase) | 1.06 (0.97-1.15) | 0.15 |
| Malignancy | 0.08 (0.008-0.76) | 0.02 |
| BMI, kg/m^2^ | 1.06 (0.98-1.15) | 0.13 |

Fixed variables were included only if they showed a p-value less than 0.2 in the previous model.

**Figure S1:** Effect of centre in the probability of intubation after HFNO. The vertical line depicts the common intercept. Horizontal bars represent 95% confidence interval for each centre.

**
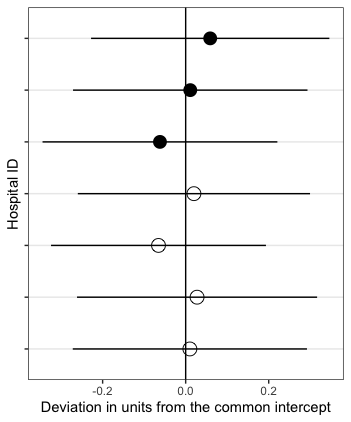
**

**Figure S2.** Histogram depicting the optimism for each of the 500 models derived in the bootstrapped samples and later validated in the whole cohort.


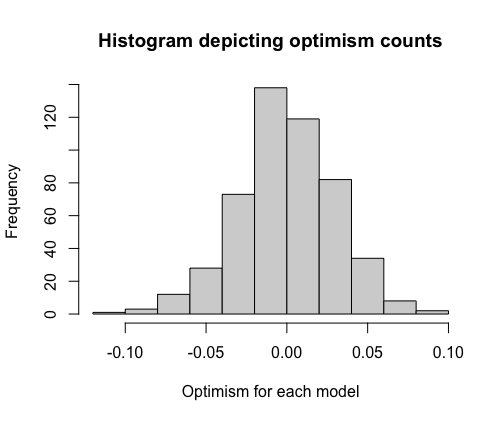


**REFERENCES:**

1. Harrell FE. *Regression Modeling Strategies: With Applications to Linear Models, Logistic and Ordinal Regression, and Survival Analysis*. (Springer, ed.).; 2015. doi:DOI 10.1007/978-3-319-19425-7
